# Effects of Lactic Acid Bacterial Inoculants on Fermentation Quality, Bacterial Community, and Mycotoxins of Alfalfa Silage under Vacuum or Nonvacuum Treatment

**DOI:** 10.3390/microorganisms9122614

**Published:** 2021-12-17

**Authors:** Xiaomiao Fan, Shanshan Zhao, Fengyuan Yang, Yuan Wang, Yanping Wang

**Affiliations:** 1 Henan Key Laboratory of Ion Beam Bio-Engineering, College of Physics, Zhengzhou University, Zhengzhou 450000, China; fanxiaomiao1125@163.com (X.F.); zsszd@gs.zzu.edu.cn (S.Z.); yangfy@gs.zzu.edu.cn (F.Y.); wangyuan5@163.com (Y.W.); 2 Henan Key Laboratory of Ion Beam Bio-Engineering, School of Agricultural Science, Zhengzhou University, Zhengzhou 450000, China; 3 State Key Laboratory of Cotton Biology, School of Agricultural Science, Zhengzhou University, Zhengzhou 450000, China

**Keywords:** alfalfa silage, vacuum treatment, nonvacuum treatment, fermentation quality, inoculants, microbial community, mycotoxins

## Abstract

To investigate the effects of lactic acid bacterial (LAB) inoculants and vacuuming on the fermentation quality and bacterial community, alfalfas were ensiled with or without a commercial LAB YX or *Lactobacillus plantarum* strain ZZUA493 for 10, 30, 60, and 90 days while undergoing either vacuum (V) or nonvacuum (NV) treatment. At 90 days, analysis of the microbial community by high-throughput sequencing was performed, and contents of aflatoxin B1 and deoxynivalenol (DON) mycotoxins in alfalfa silage were determined. In all inoculated alfalfa silage, irrespective of V or NV treatment, lactic acid (LA) content increased, pH (*p* < 0.05), and ammonia nitrogen (*p* < 0.05) content decreased, and no butyric acid was detected. *Lactobacillus* or *Pediococcus* became the dominant genus, and the abundance of *Garciella* decreased in alfalfa silage with the addition of either inoculant. The LAB inoculants YX and ZZUA493 helped reduce the mycotoxin content in alfalfa silage. The abundance of *Garciella* in the control and DON content in all alfalfa silage groups were higher (*p* < 0.05) in NV than V. In summary, LAB inoculants and vacuuming had a positive influence on alfalfa silage quality, and LAB inoculants were effective in reducing mycotoxins in silage alfalfa.

## 1. Introduction

Alfalfa is a perennial herbaceous plant cultivated widely in humid and rainy southern China, characterized by high crude protein, nutritional value, and digestibility [1]. Ensiling is a conservation method for preserving green forage crops, which takes advantage of simple technology, improves feed palatability, and extends the storage time [2,3] and, due to the seasonal harvest of alfalfa, is necessary to preserve it to cope with the shortage of winter livestock food. Alfalfa is often difficult to ensile because of its high buffering capacity and low water-soluble carbohydrate (WSC) content [4], hindering successful fermentation. Adding lactic acid bacteria (LAB) inoculant has become an effective measure to enhance the fermentation quality of alfalfa silage.

Silage fermentation occurs under anaerobic conditions by forming an acidic environment by epiphytic LAB [5]. These can convert WSC into organic acids, mainly lactic acid (LA), and promote the reduction in pH, effectively preventing putrefaction of the alfalfa silage and inhibiting the growth of undesirable microorganisms [6,7]. Some research has focused on enhancing alfalfa fermentation indicators by LAB additives, such as reduction of ammonia nitrogen (NH_3_-N) and pH and the increase in LA content [8,9]. The fermentation quality of silage largely depends on the composition of the microbial community [10]; the LAB commonly used to improve the fermentation quality of silage include *Lactobacillus*, *Pediococcus*, and *Enterococcus* [11]. In the initial stages of ensiling, cocci, including *Leuconostoc*, *Enterococcus*, *Streptococcus*, and *Lactococcus,* thrive and promote LA fermentation, while *Lactobacillus* plays a leading role later [7,12]. Some studies reported that *Pediococcus pentosaceus* inoculation could improve silage fermentation by decreasing pH and increasing LA content and effectively inhibit the proliferation of *Monascus* [13]. As the most used bacterial inoculum, *Lactobacillus plantarum* has strong acidification properties, which are conducive to inhibiting competitive microorganisms and effectively improving the fermentation quality of silage.

Some undesirable factors can also lead to aerobic decay of the ensiled matter, including insufficient dry matter (DM) concentration at harvest, loose compaction, air infiltration and delayed pH reduction [14,15]. Previous studies have shown that low-density sealing and insufficient compaction may lead to an increase in silage temperature and the growth of undesirable microorganisms [10,15]. The air in alfalfa silage is usually expelled using a vacuum sealer to avoid feed spoilage. In this study, silage samples were treated with a vacuum (V) and nonvacuum (NV), in which NV treatment simulated poor silage conditions, such as insufficient compaction.

Mycotoxins are frequently present in silage, and consuming mycotoxins is detrimental to livestock health, with the most frequent mycotoxins in silage including aflatoxin B1 (AFB1), deoxynivalenol (DON), fumonisins, and ochratoxin A. Contamination with mycotoxins can lead to adverse effects such as reduced animal feed intake, liver function, and production performance. They can also be carcinogenic, teratogenic, and lethal and cause significant economic losses. Among them, AFB1, the major toxin of toxigenic *Aspergillus species*, is the most toxic and carcinogenic [16].

Feed contaminated with multiple mycotoxins may have interactive effects on livestock [17], and it is, therefore, essential to degrade or transform them. Some LAB can reduce mycotoxin concentrations in silage during fermentation by binding and degradation [18,19,20]. Silage inadequately sealed and insufficiently compacted to maintain good anaerobic conditions will allow mould proliferation and mycotoxin production [20]. In the presence of oxygen, storage fungi such as *Aspergillus* and *Penicillium* can grow and produce additional mycotoxins even in well-preserved silage [18].

This study aimed to explore the effects of inoculation with or without LAB inoculants with V and NV treatment on the bacterial community, fermentation quality and mycotoxins of alfalfa silage.

## 2. Materials and Methods

### 2.1. Silage Making

Alfalfa was cultivated in Zhengzhou, Henan Province, which has a temperate monsoon climate and is located at 34.76° N, 113.65° E, at an altitude of 110.4 m above sea level. Alfalfa at the early bloom stage from the second cutting was harvested and wilted for 24 h. After wilting, alfalfa was chopped into 1–2 cm lengths using a crop chopper. The experimental treatments were V treatment or NV treatment. Within these were three treatment groups, including no additive using distilled water as a control (CK), use of one commercial LAB inoculant YX of 1 × 10^6^ colony forming units (cfu)/g, and use of the selected *Lactobacillus plantarum* strain ZZUA493 at 1 × 10^6^ cfu/g (hereafter abbreviated to 493). Among them, YX was isolated from the Yaxin alfalfa ensiling additive (Yaxin Biotechnology Co., Ltd., Taiwan, China), and 493 was isolated from silage alfalfa in Hebei Province, China. Each of the additives was mixed homogenously with the wilted alfalfa, then 500 g of chopped alfalfa material was packed manually into a plastic bag (Dongda, Zhengzhou, China). One group was vacuum sealed tightly using a P-290 sealer (Shineye, Dongguan, China), and the other group was sealed without vacuuming. A total of 72 bags were prepared, comprising two groups of three treatments with three replicates stored over four periods, and kept at an ambient temperature of 20–35 °C. The periods were 10, 30, 60, and 90 days. After every ensiling period, the bags were opened on a clean bench (Jiangsu Sujing Group Co., Ltd., Suzhou, China), and samples were saved for later determination of fermentation indices and chemical composition. After 90 days of ensilage, the microbial community was analyzed using high-throughput sequencing, and two mycotoxin contents in alfalfa silage were determined.

### 2.2. Chemical Composition and Fermentation Quality Analyses

The DM content of subsamples was measured immediately by the oven (Shanghai Jing Hong Laboratory Instrument Co., Ltd., Shanghai, China) drying at 65 °C for 48 h. The oven-dried samples were ground to pass a 1 mm sieve for later chemical analysis. The WSC content was quantified using anthrone colourimetry [21]. Ten-gram samples of ensiled alfalfa were thoroughly mixed with 90 mL of sterilized deionized water, and pH was measured with a pH meter (Mettler Toledo Co., Ltd, Greifensee, Switzerland). The resulting filtrates from the samples were filtered through four layers of cheesecloth and used for determining NH_3_-N and organic acids content. The content of NH_3_-N was determined using the phenol-hypochlorite procedure [22]. Organic acids including LA, butyric acid (BA), propionic acid (PA), and acetic acid (AA) were quantified by HPLC system (Waters Inc., Milford, MA, USA; column: Carbomix H-NP 10:8% (7.8 × 300 mm × 10 µm), Sepax Technologies, Inc., Santa Clara, CA, USA; Waters Inc.; mobile phase: 0.0254% H_2_SO_4_; flow rate: 0.6 mL min^−1^; detector: UV detector; temperature: 55 °C; wavelength: 214 nm; injection volume: 10 µL; retention time: 22 min) [23]. The concentration of each standard solution in the standard solution was: LA, 30 mg/kg; AA, 30 mg/kg; PA, 20 mg/kg; and BA 20 mg/kg. Each was then diluted into five gradients, the concentrations of LA and AA after dilution were 3 mg/kg, 1.50 mg/kg, 0.75 mg/kg, 0.375 mg/kg, and 0.1875 mg/kg respectively The concentrations of PA and BA were 2 mg/kg, 1 mg/kg, 0.2 mg/kg, 0.25 mg/kg, and 0.125 mg/kg respectively.

### 2.3. Bacterial Community Analysis

Each 10 g sample was mixed with 90 mL of sterile phosphate buffer (Tianjin Hengxing chemical reagent manufacturing Co., Ltd., Tianjin, China) and shaken in a 180-rpm shaker (Changzhou Putian Instrument Manufacturing Co., Ltd., Changzhou, China) for 1 h. The mixture was filtered with four layers of sterile gauze and centrifuged at 4 °C for 15 min at 8000× *g* using a centrifuge (Eppendorf AG, Hamburg, Germany). The sediment was mixed with 1 mL of sterile phosphate-buffered saline. The precipitates were collected by centrifugation at 15,000× *g* at 4 °C for 5 min. Total DNA extraction was performed using a Bacterial DNA Kit D3350-02 (Omega Biotek, Norcross, GA, USA) according to the manufacturer’s instructions. The concentrations of extracted DNA samples were determined using a NanoDrop ND-2000 spectrophotometer (Thermo Fisher Scientific Inc., Carlsbad, CA, USA), and the quality of the extracted DNA was assessed using 1% agarose gel electrophoresis. The DNA samples were subjected to PCR amplification with the forward Primer F (Illumina adapter sequence 1 + CCTACGGGNGGCWGCAG) and the reverse Primer R (Illumina adapter sequence 2 + GACTACHVGGGTATCTAATCC) targeting the V3–V4 regions of the bacterial 16SrRNA gene (approximately 460 bp). *Escherichia coli* CMCC (B) 44102 (NRRL accession No. B-1109) was a positive control to confirm that the procedure correctly assessed this identification and that the primers used worked normally. Amplicons were separated and purified from 2% agarose gel using Agencourt AMpure XP nucleic acid purification magnetic beads to obtain an original library of samples. The DNA samples were sequenced using a Miseq platform (Genesky Bio-Tech Co., Ltd., Shanghai, China). All paired reads were processed using FLASH2 (v.1.2.11) and filtered using the QIIME quality control process (v.1.9.1) to obtain high-quality sequences with quality scores above 80. The alpha diversities of samples were computed using Mothur software (v.1.9.1), including the Good’s coverage, Shannon diversity index, and Chao1 richness estimator. The beta diversity analyses were conducted using the R software package (v.2.15.3). The sequence data had been submitted to the NCBI database under accession number PRJNA776111.

### 2.4. Determination of AFB1 and DON in Alfalfa Silage

The mycotoxin concentrations in samples were determined by direct competitive ELISA. The oven-dried, ground samples of alfalfa silage were obtained as previously described. Of 70% *v/v* methanol (Tianjin Zhiyuan Chemical Reagent Co., Ltd., Tianjin, China), 10 mL was added to 5 g of each oven-dried ground sample. After the mixture was thoroughly mixed by vigorous shaking and filtered through a filter paper, the eluent was collected. The AFB1 and DON concentrations were determined using the AFB1 ELISA and DON ELISA Kits (Shanghai Lian Shuo Biotechnology Co. Ltd., Shanghai, China), according to the manufacturer’s instructions. Absorbance was measured at 450 nm using a multimode reader (Thermo Fisher Scientific Inc., Carlsbad, CA, USA). A standard curve was drawn using external standards for each mycotoxin provided with the kit and then used to calculate mycotoxin concentrations. The detection limit of the kits was 0.05 ng/mL and 0.95 μg/mL for AFB1 and DON, respectively. The limit of quantification was 0.1 ng/mL and 2 μg/mL for AFB1 and DON, respectively.

### 2.5. Statistical Analysis

All statistical procedures were performed using Statistical Packages for the Social Sciences software (v.21.0; SPSS Inc., Chicago, IL, USA) and Excel software (Microsoft Corporation, Redmond, WA, USA). The data on fermentation quality and microbial compositions of alfalfa silage were analyzed by two-way ANOVA. One-way ANOVA was used to evaluate the effect of different treatments on mycotoxin concentrations in silage alfalfa. Tukey’s multiple comparison method was used to determine statistical differences between means. Significance was defined at *p* < 0.05, and high significance was defined as *p* < 0.01.

## 3. Results

### 3.1. Forage Characteristics before Ensiling 

The DM of pre-ensiled alfalfa was 39.65%. The pre-ensiled alfalfa had a WSC content of 5.13 g/kg DM and a pH of 6.79. Neither NH_3_-N nor organic acids were detected in pre-ensiled alfalfa.

### 3.2. Chemical Composition and Fermentation Characteristics of Alfalfa Silage

The chemical composition of alfalfa with different treatments is shown in Table 1. The DM content of alfalfa did not show significant differences between treatments and CK (Table 1). Over the 10 days of ensiling, whether V or NV, the WSC content in the CK was lower than in the two inoculated groups but was significantly lower in V-CK than V-YX (*p* < 0.05).

The fermentation characteristics of alfalfa for different treatments are shown in Table 2. The LA content of V-CK was lower than that of V-YX and V-493 at each silage stage and significantly lower at 10 and 90 days (*p* < 0.05). The LA content in NV-CK was also lower than that of NV-YX and NV-493 at each silage stage and significantly lower at 30 and 60 days (*p* < 0.05). At 90 days, the LA contents of NV-YX and NV-493 were significantly lower than those of V-YX and V-493, respectively (*p* < 0.05).

The AA content accumulated with ensiling duration and was lower in NV-CK than V-CK (Table 2). The silages treated with inoculants had lower pH and NH_3_-N content than CK during each silage period irrespective of V treatment (*p* < 0.05). Compared with 30 and 60 days, the content of NH_3_-N in V-CK and NV-CK increased significantly at 90 days of silage, especially in NV-CK (*p* < 0.05). For 90 day silage, PA content in V-CK and NV-CK was 13.11 and 12.44 g/kg DM, respectively. The BA content was higher in NV-CK than V-CK silage with 12.17 and 11.53 g/kg DM, respectively. 

### 3.3. Bacterial Diversity and Community Analysis of Alfalfa Silage at 90 Days 

#### 3.3.1. Alpha and Beta Diversity Indices of Bacterial Communities

The alpha diversities of bacterial communities in alfalfa silage are presented in Table 3. The average Good’s coverage (> 99%) indicated that the sequencing depth was sufficient to capture most bacterial communities in all silages. The Shannon index was used to evaluate bacterial diversities directly and ranged within 0.36–1.73 (Table 3), while the Chao1 index demonstrated the richness of the bacterial community with a range of 254.22–371.37. At 90 days, the bacterial diversity of V-CK was significantly higher than that of V-YX and V-493 (*p* < 0.05). The bacterial diversities of V-YX and NV-YX were significantly lower than those of V-493 and NV-493, respectively (*p* < 0.05). The diversity of NV-CK was significantly lower than that of V-CK (*p* < 0.05), and the diversity of NV-493 was significantly higher than that of V-493 (*p* < 0.05). The Chao1 index showed that the richness of the bacterial community was not significantly changed by adding either inoculant compared with CK.

**Figure 1:**
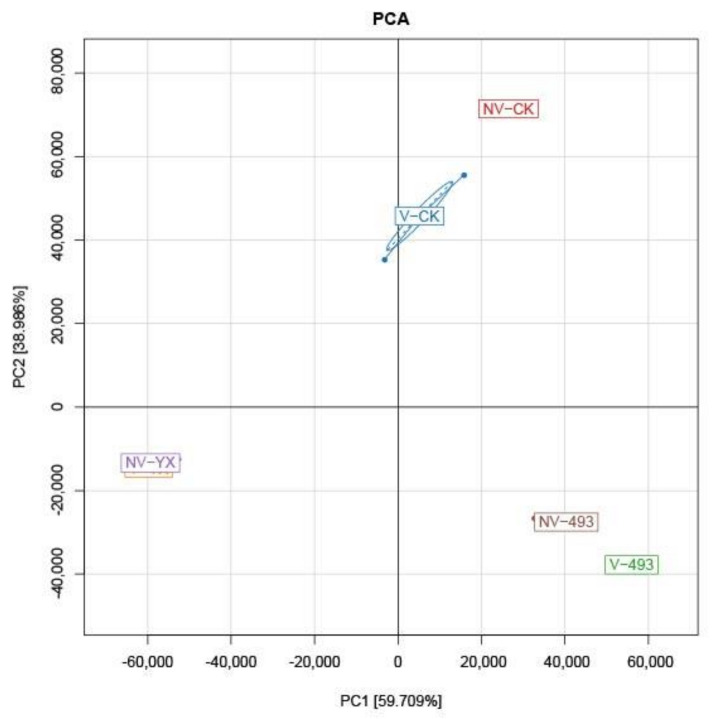
Principal component analysis (PCA) of bacterial communities in alfalfa silage at 90 days. Control, CK; vacuum treatment, V; inoculated with YX, YX; inoculated with *Lactobacillus plantarum* ZZUA493, 493; nonvacuum treatment, NV.

The variation of the bacterial community was further explained by the analysis of beta diversity indices. Principal component analysis (PCA) clearly reflected the change of microbial community, with principal components 1 and 2 explaining 59.71% and 38.99% of the total variance, respectively (Figure 1). There was a clear separation between CK and silages treated with inoculants, indicating differences in the microbial communities. Compared with V-CK, the distribution of the bacterial community within NV-CK was more clustered at 90 days and the silages treated with the two inoculants were separate from each other. The distance between V-YX and NV-YX silages was very close, while V-493 was distant from NV-493.

#### 3.3.2. Abundance of Bacterial Community

**Figure 2:**
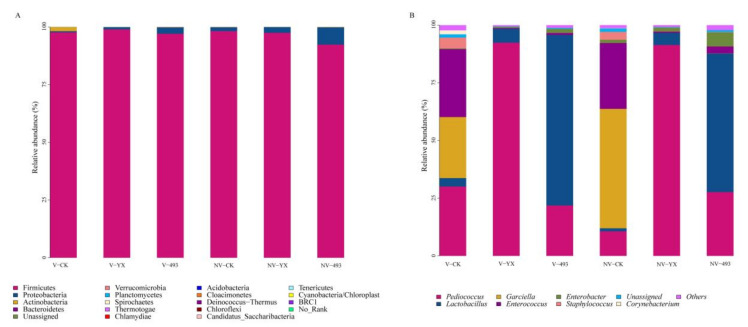
Relative abundance of bacteria at phylum (**A**) level and genus (**B**) level of alfalfa after 90 days of ensiling. Control, CK; vacuum treatment, V; inoculated with YX, YX; inoculated with *Lactobacillus plantarum* ZZUA493, 493; nonvacuum treatment, NV.

The phylum-level relative abundance of the bacterial community in alfalfa silage at 90 days is shown in Figure 2A. At 92.36–98.96%, Firmicutes was the predominant phylum in all silages, representing most complete sequences. In the three treatment groups, the relative abundance of Firmicutes was lower in silages treated with NV compared to V. At the genus level (Figure 2B), compared with V-CK, NV-CK showed a higher relative abundance of *Garciella* (*p* < 0.01) but lower relative abundances of *Pediococcus* and *Lactobacillus* at 90 days. The prevalent genera in V-CK were *Pediococcus* (30.06%), *Enterococcus* (29.43%) and *Garciella* (26.43%), as well as *Staphylococcus* (4.69%), *Lactobacillus* (3.67%) and *Corynebacterium* (1.74%). The dominant genera in NV-CK were *Garciella* (51.73%), *Enterococcus* (28.55%) and *Pediococcus* (10.67%), followed by *Staphylococcus* (3.43%), *Enterobacter* (1.41%) and *Lactobacillus* (1.31%). Whether V or NV treatment, the addition of YX or 493 inhibited the growth of *Staphylococcus*. Compared with CK, the relative abundances of *Pediococcus* or *Lactobacillus* were increased by adding YX or 493. *Pediococcus* exhibited high abundance in both V-YX (92.39%) and NV-YX (91.39%) silages, with a higher relative abundance of 1.72% of *Enterobacter* in NV-YX compared with 0.51% in V-YX silage (*p* < 0.01). *Lactobacillus* became the predominant genus both in V-493 and NV-493 silages, and there was a lower relative abundance of *Lactobacillus* in NV-493 than V-493 silage (60.06% vs. 73.90%, *p* < 0.01). There was a higher relative abundance of *Enterobacter* in NV-493 than V-493 silage (6.13% vs. 1.77%, *p* < 0.01).

**Figure 3:**
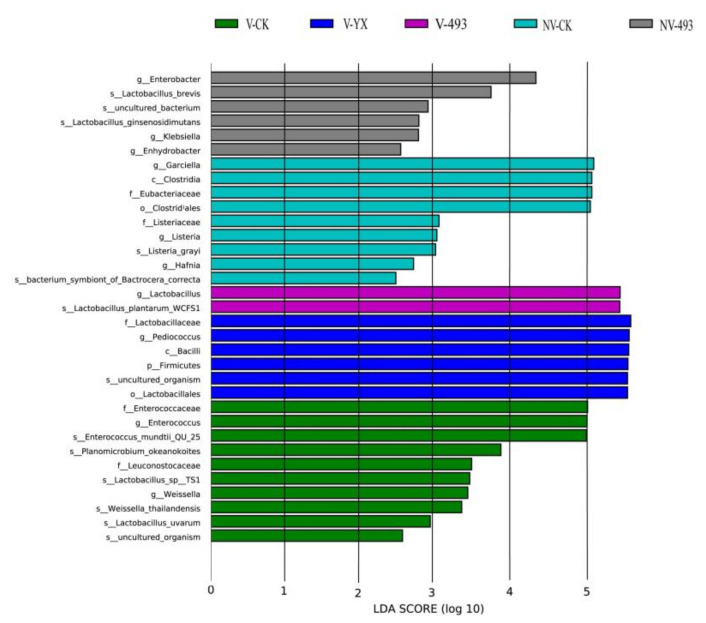
Comparison of bacterial variations using LEfSe analysis. Control, CK; vacuum treatment, V; inoculated with YX, YX; inoculated with *Lactobacillus plantarum* ZZUA493, 493; nonvacuum treatment, NV.

Linear discriminant analysis effect size (LEfSe) analysis was used to further illustrate the variations in bacterial communities among the groups (Figure 3). *Enterococcus mundtii* was significantly higher in V-CK. Some putrefactive and pathogenic genera such as *Garciella* and *Listeria* were higher in NV-CK. The genus *Pediococcus* was higher in V-YX. The relative abundance of *Lactobacillus plantarum* was lower and that of *Enterobacter* markedly higher in NV-493. The relative abundances of *Lactobacillus*, *Garciella* and *Enterobacter* were markedly correlated with V treatment, inoculants and their interactions (*p* < 0.05, Table 4).

#### 3.3.3. Correlation Analysis between Bacterial Community and Fermentation Characteristics of Alfalfa Silage at 90 Days

Mantel tests were used to reveal the correlation between the relative abundance of bacterial community at the genus level and the fermentation characteristics of alfalfa silage at 90 days. Spearman’s correlation analysis showed that the Shannon index was positively correlated with pH value, NH_3_-N, and BA (*p* < 0.05, Table 5). The abundance of the bacterial community was positively correlated with LA concentration and negatively correlated with WSC (*p* < 0.05). *Lactobacillus* was negatively correlated with pH and BA and positively correlated with AA and WSC (*p* < 0.05). *Pediococcus* was negatively correlated with pH and NH_3_-N (*p* < 0.05). *Garciella*, *Staphylococcus*, and *Enterococcus* were positively correlated with pH, NH_3_-N, and BA (*p* < 0.05).

### 3.4. Effects of Treatments on AFB1 and DON Contents in Alfalfa Silage at 90 Days

**Figure 4:**
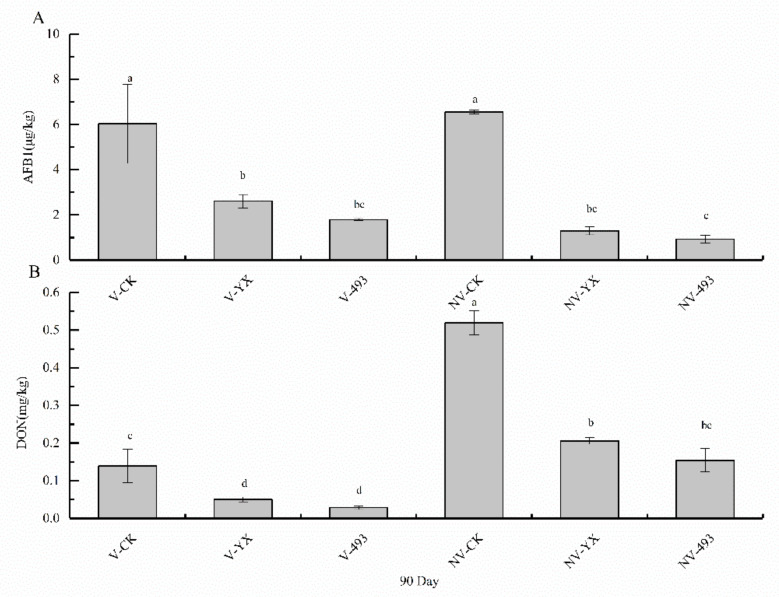
AFB1 (**A**) and DON (**B**) contents of alfalfa silage with different treatments at 90 days. CK, control; V, vacuum treatment; YX, inoculated with YX; 493, inoculated with *Lactobacillus plantarum* ZZUA493; NV, nonvacuum treatment. Different lowercase letters indicate a significant difference between samples (*p* < 0.05).

Irrespective of V treatment, the contents of mycotoxins AFB1 and DON in alfalfa inoculated with YX or 493 were all significantly lower than those of CK at 90 days (*p* < 0.05), and these two mycotoxin levels were lower in alfalfa inoculated with 493 than with YX (*p* > 0.05, Figure 4). There was no significant difference in AFB1 content between V- and NV-treated alfalfa silage for 90 days in either CK or the two inoculated groups (*p* > 0.05, Figure 4A). The DON contents in NV-CK, NV-YX, and NV-493 were higher than those in V-CK, V-YX, and V-493, respectively, at 90 days (*p* < 0.05, Figure 4B).

## 4. Discussion

### 4.1. Characteristics of Pre-Ensiled Alfalfa

The fermentation quality of silage was affected by feed characteristics [24]. As a conventional technique to improve the fermentation quality of silage, wilting can increase DM content and reduce the effluent loss in the ensiling process, which may also have a major impact on the bacterial community during fermentation [7,25]. The DM content of forage has a significant influence on silage fermentation, feed intake and production performance [26]. In this study, the DM content of pre-ensiled alfalfa was wilted to 39.65%, which achieved the recommended level [25]. The WSC content in pre-ensiled alfalfa was also a key factor affecting fermentation quality, in which sufficient WSC content exceeding 60 g/kg DM was conducive to alfalfa fermentation, producing LA to reduce pH and form an acidic silage environment [3]. The WSC in alfalfa is low, and this is exacerbated after wilting [23]. In this study, the WSC content of 5.13 g/kg DM in pre-ensiled alfalfa was lower than the threshold for well-preserved silage. The low WSC content is one reason why alfalfa is difficult to ensile, and the LAB inoculant was added was to make it the dominant bacteria, reducing the availability of WSC to other harmful bacteria.

### 4.2. Effect of LAB Inoculants and V Treatment on Fermentation Characteristics of Alfalfa Silage

The two LAB inoculants and V treatment had significant positive effects on fermentation characteristics. Compared with pre-ensiled alfalfa, the WSC content of all treatments decreased after ensiling. At ten days, the WSC content in CK was lower than in both inoculated groups, possibly due to the miscellaneous bacteria in CK consuming more WSC for growth and reproduction. Silage pH is an important parameter affecting fermentation quality and is closely related to the LA concentration of raw materials [27,28]. These two inoculants effectively promoted LA fermentation and AA accumulation. As the most potent acid in silage, LA reduces pH more effectively, so silage quality is enhanced by increasing the LA content and inhibiting the growth of adverse bacteria and fungi [29,30]. Compared with CK, whether V or NV, both LAB inoculants increased LA content and decreased pH, suggesting they could improve silage fermentation quality. Previous reports have shown that LAB inoculants have similar effects on silage feed, but V and NV treatments have rarely been used in such studies [4,12]. In this study, at 90 days, the LA contents in NV-YX and NV-493 were significantly higher than in NV-CK, and the LA contents in NV-YX and NV-493 were significantly lower than in V-YX and V-493, respectively. The V treatment was more conducive to fermentation and LA accumulation, showing the importance of maintaining anaerobic conditions during silage. Kung et al. [31] observed that the LA content was lower in low density compared with normal density packing (176 vs. 240 kg DM/m^3^), and the inoculation of LAB even at low-density packing was still beneficial to the fermentation quality of corn silage. In this study, LA content in V-CK and NV-CK decreased significantly during 60–90 days of ensiling, possibly related to the utilization of LA by *Clostridium* and other harmful bacteria [2]. The LA content decreased for 90 day compared with 60 day silage in NV-493, which could be due to higher *Enterobacter* concentration, as LA can be utilized by *Enterobacter* [32].

The NH_3_-N content is a reliable indicator of protein degradation [33]. During the ensiling process, NH_3_-N accumulation can be explained by the activity of plant enzymes and fermentation by *Clostridium* and *Enterobacter* [30]. In this study, silage inoculated with either LAB additive had lower NH_3_-N levels than CK. This may be due to LAB inoculation because the acidic substances produced by LAB fermentation rapidly lower pH, and the bacteriostatic and bactericidal effects of organic acids reduce fermentation by *Clostridium* [34], which is recognized as a typical cause of NH_3_-N accumulation [23]. This is consistent with the results of Wang et al. [35], who found that inoculating one commercial inoculant of *Lactobacillus plantarum* and three isolated LAB strains significantly decreased the NH_3_-N levels compared with controls both in Italian ryegrass and oat silages. The higher content of NH_3_-N in CK in our study was related to the higher abundance of pathogenic bacteria *Staphylococcus* and *Corynebacterium*. No significant differences in NH_3_-N content were observed between V-493 and NV-493 or between V-YX and NV-YX. Similarly, Zhang et al. [36] found no significant difference in NH_3_-N content between two silage density levels when the isolated strains were added.

The existence of BA in silage results from undesired Clostridia fermentation, which would reduce the intake of livestock [27] and this was detected in both V-CK and NV-CK and was related to their high levels of *Garciella*. At 90 days, the PA levels of 13.11 and 12.44 g/kg DM for V-CK and NV-CK silages, respectively, exceeded the acceptable range for this acid which should be 1–10 g/kg DM for better quality silage [23].

### 4.3. Effect of LAB Inoculants and V Treatment on Bacterial Community of Alfalfa Silage at 90 Days 

Adding the LAB inoculations to silage resulted in lower bacterial diversity for V-CK and NV-CK alfalfa silages as indicated by lower Shannon indices (Table 3). Low pH was the main factor limiting microbial diversity because the growth of many aerobic epiphytic bacteria is inhibited by an acidic environment [4,37,38,39]. The reason that the bacterial diversity of NV-CK was significantly lower than that of V-CK might be the higher relative abundance of the predominant genus of *Garciella* in NV-CK. The relative abundance of the dominant genus *Pediococcus* in V-YX and NV-YX exceeded 90%, which may be why they had significantly lower bacterial diversity than other treatments. The higher the abundance of dominant bacteria, the lower is the diversity of the microbial community [40]. The bacterial diversity of NV-493 was higher than that of V-493 silage, possibly because the growth of *Enterobacter* in NV-493 was more vigorous than in V-493, and there was a lower relative abundance of the dominant genus *Lactobacillus* in NV-493.

The reason for Firmicutes being the dominant phylum is that most bacteria involved in LA fermentation during ensiling are of this phylum rather than Proteobacteria [41]. The dominant bacteria of V-493 and NV-493 were *Lactobacillus*, and the relative abundance of *Lactobacillus* was lower in NV-493 than V-493 because NV treatment was more conducive to the growth of aerobic microorganisms. As a dominant genus, *Lactobacillus* plays a significant role in fermentation quality because it can produce LA, reduce pH, and inhibit the growth of undesirable bacteria [42]. *Garciella* belongs to Clostridia, were present in V-CK and NV-CK and reached higher levels in the NV-CK silage, which is considered undesirable. The harmful genus *Garciella* was effectively inhibited by adding either inoculant and NV treatment increased *Enterobacter* in alfalfa silage, as seen in NV-493 (6.13%), V-493 (0.77%), NV-YX (1.73%), and V-YX (0.51%). To metabolize LA into BA, *Garciella* can cause protein degradation, NH_3_-N production, and DM loss [27,43]. *Enterobacter* are also considered undesirable bacteria in silage because they can compete with LAB for available WSC and metabolize LA into AA and other products [3,42]. *Enterobacter* are aerobic bacteria and can increase their growth and reproduction under NV treatment, resulting in higher relative abundance and consuming more fermentable sugars. Previous research also found that poor compaction led to more bacilli and lower fermentation end-products [31]. Inoculation of LAB could inhibit the growth of potentially pathogenic bacteria such as *Staphylococcus* and improve feed safety.

### 4.4. Correlation Analysis between Bacterial Community and Fermentation Characteristics

Mantel tests indicated that fermentation characteristics were closely related to bacterial community composition. The pH was positively correlated with the bacterial community diversity. At 90 days, regardless of V, silage inoculated with YX and 493 had lower pH and bacterial diversity than those of CK. The reason may be that inoculation of YX and 493 made *Pediococcus* and *Lactobacillus* the dominant genera, which inhibited the growth of other bacteria under acidic conditions. The shift in the bacteria community could also explain the lower NH_3_-N when silage was inoculated by both types of LAB. The WSC was negatively correlated with bacterial community richness, as previously reported by Yang et al. [38] because WSC was consumed during microbial growth.

Correlation analyses indicated that *Lactobacillus* and *Pediococcus* were positively correlated with fermentation quality because most of the LAB (e.g. *Lactobacillus*, *Pediococcus*, *Lactococcus*, and *Weissella*) are conducive to LA fermentation [37]. At 90 days, the LA content in NV-493 was significantly lower than that in V-493, corresponding to the low relative abundance of *Lactobacillus* in NV-493. The high LAB abundance promoted the full production of organic acids, which reduced the silage pH, inhibiting proteolysis activity, the growth of adverse microorganisms, and the formation of NH_3_-N. *Garciella*, *Enterococcus* and *Staphylococcus* were negatively correlated with fermentation quality (Table 5). These genera were distributed in the V-CK and NV-CK groups, and the relative abundance of *Garciella* in NV-CK was significantly higher than in V-CK (*p* < 0.05), which could produce BA by utilizing organic acids as fermentation substrates [43]. The higher pH in V-CK and NV-CK silages may benefit *Garciella* growth and protein hydrolysis. At lower pH, the proteolytic activity of silage decreases [36], and higher pH could explain the higher NH_3_-N content in V-CK and NV-CK. The presence of BA and NH_3_-N in silage is undesirable. *Enterococcus* is a common LA-producing bacteria in silage and is widely used to improve fermentation quality [27]. Although *Enterococcus* was present in V-CK and NV-CK, it did not become the dominant genus, and the resulting poor fermentation quality was associated with high *Garciella* abundance.

### 4.5. Effect of Two LAB Inoculants and V Treatment on AFB1 and DON Contents of Alfalfa Silage

The legal limit of AFB1 is 10 μg/kg and of DON is 1 mg/kg in Chinese feed products. In this study, the AFB1 and DON contents in all 90 day silage samples did not exceed the legal limits. Previous studies have shown that some LAB can degrade or immobilize mycotoxins during silage fermentation by combining with the surface of mycotoxins [18,19]. Antifungal compounds such as organic acids, carboxylic acids, and phenolic compounds produced by LAB can also reduce mycotoxins produced by moulds [20]. At 90 days, regardless of V treatment, the use of YX or 493 decreased AFB1 and DON levels in alfalfa silage compared with CK, with 493 being most effective at reducing mycotoxins. Silage conditions are not conducive to *Fusarium* (i.e., fungal genus) because they cannot tolerate low pH and anaerobic conditions [16]. The DON present is usually produced by *Fusarium,* and its content was higher in CK. The lower DON content in silage after inoculation of either LAB additive might be related to the lower pH in the silage environment. In this study, NV treatment was conducive to the accumulation of DON mycotoxins at 90 days, so increasing the anaerobic environment in alfalfa silage by V treatment reduced the DON content.

## 5. Conclusions

This study confirmed that inoculation of alfalfa silage with YX and 493 improved fermentation quality under V or NV treatment. With more significant improvement for V. *Garciella* was inhibited, while *Pediococcus* and *Lactobacillus* were enhanced by the two LAB inoculants. In CK, the relative abundance of *Garciella* in alfalfa silage was higher in NV than V treatment. The results revealed that both inoculants were helpful in reducing AFB1 and DON contents of alfalfa silage under V or NV treatment. The NV treatment was instrumental in the accumulation of DON in alfalfa silage.

In summary, the two LAB inoculants and the V treatment had positive effects on alfalfa silage quality, and LAB inoculation effectively reduced mycotoxin levels.

## Figures and Tables

**Table 1:** Effects of vacuum treatment and inoculants on chemical compositions in alfalfa silage at different silage opening times.

Items	Treatments	Ensiling Days (d)				SEM	*p*-Value		
		10	30	60	90		T	D	T × D
DM (g/kg FM)	V-CK	40.08	40.02	39.84	40.12	0.14	NS	NS	NS
	V-YX	40.38	40.63	40.30	40.60				
	V-493	40.06	39.95	40.45	40.25				
	NV-CK	40.34	40.09	41.12	40.44				
	NV-YX	39.15	39.77	40.10	40.26				
	NV-493	39.82	39.71	40.44	39.73				
WSC (g/kg DM)	V-CK	1.41 ^B^	1.11	1.09	1.10	0.10	<0.01	<0.01	<0.05
	V-YX	1.92 ^Aa^	1.41 ^b^	1.48 ^b^	1.22 ^b^				
	V-493	1.85 ^ABa^	1.43 ^b^	1.31 ^b^	1.32 ^b^				
	NV-CK	1.42 ^B^	1.40	1.14	1.08				
	NV-YX	1.82 ^ABa^	1.27 ^b^	1.12 ^b^	1.17 ^b^				
	NV-493	1.72 ^ABa^	1.48 ^ab^	1.08 ^b^	1.27 ^b^				

Dry matter, DM; fresh matter, FM; water-soluble carbohydrates, WSC; control, CK; vacuum treatment, V; inoculated with YX, YX; inoculated with *Lactobacillus plantarum* ZZUA493, 493; nonvacuum treatment, NV; treatments, T; ensiling days, D; interaction between T and D, T × D; standard error of mean, SEM; not significant, NS. Means with different letters in the same row (a–b) or column (A–B) differ (*p* < 0.05).

**Table 2:** Effects of vacuum treatment and inoculants on fermentation characteristics in alfalfa silage at different silage opening times.

Items	Treatments	Ensiling Days (d)				SEM	*p*-Value		
		10	30	60	90		T	D	T × D
pH	V-CK	6.63 ^Aa^	6.56 ^Aab^	6.43 ^Ab^	6.40 ^Ab^	0.05	<0.01	<0.01	<0.01
	V-YX	5.30 ^Ba^	5.16 ^Cab^	5.12 ^Dab^	5.02 ^Cb^				
	V-493	5.47 ^Ba^	5.35 ^BCab^	5.27 ^CDb^	5.13 ^BCb^				
	NV-CK	6.65 ^Aa^	6.43 ^Ab^	6.03 ^Bc^	6.34 ^Ab^				
	NV-YX	5.48 ^Ba^	5.33 ^BCab^	5.41 ^Ca^	5.18 ^BCb^				
	NV-493	5.46 ^B^	5.39 ^B^	5.33 ^CD^	5.28 ^B^				
LA (g/kg DM)	V-CK	15.33 ^Cb^	22.23 ^Ba^	20.04 ^Bab^	13.77 ^Db^	1.05	<0.01	<0.01	<0.01
	V-YX	21.15 ^Bc^	32.89 ^Aa^	25.76 ^ABb^	31.93 ^Aa^				
	V-493	27.19 ^A^	26.99 ^AB^	28.73 ^A^	29.79 ^AB^				
	NV-CK	19.37 ^BCa^	22.93 ^Ba^	22.64 ^Ba^	12.33 ^Db^				
	NV-YX	21.51 ^Bb^	28.63 ^Aa^	24.09 ^Bb^	26.75 ^Bab^				
	NV-493	25.07 ^ABab^	28.35 ^Aa^	26.12 ^ABa^	21.69 ^Cb^				
AA (g/kg DM)	V-CK	13.88 ^b^	18.01 ^a^	19.48 ^ABa^	18.41 ^Ba^	1.06	<0.01	<0.05	<0.05
	V-YX	12.29 ^c^	16.99 ^b^	21.82 ^Aa^	23.67 ^Aa^				
	V-493	13.10 ^c^	17.44 ^b^	20.61 ^ABab^	22.54 ^ABa^				
	NV-CK	12.88	15.34	16.64 ^B^	15.73 ^B^				
	NV-YX	12.77 ^b^	16.93 ^a^	21.44 ^Aa^	21.37 ^ABa^				
	NV-493	11.68 ^b^	17.99 ^a^	20.07 ^ABa^	20.93 ^ABa^				
LA/AA	V-CK	1.19 ^Ba^	1.24 ^Ba^	1.03 ^ab^	0.76 ^Bb^	0.14	<0.05	<0.05	<0.05
	V-YX	1.72 ^ABab^	1.84 ^Aa^	1.20 ^b^	1.37 ^Ab^				
	V-493	2.01 ^Aa^	1.55 ^ABb^	1.28 ^b^	1.33 ^Ab^				
	NV-CK	1.50 ^Ba^	1.51 ^ABa^	1.36 ^a^	0.79 ^Bb^				
	NV-YX	1.52 ^Bab^	1.55 ^ABa^	1.14 ^b^	1.26 ^Aab^				
	NV-493	2.15 ^Aa^	1.58 ^ABb^	1.23 ^bc^	1.04 ^ABc^				
NH_3_-N (g/kg DM)	V-CK	4.24 ^Ac^	6.05 ^Ab^	6.50 ^Ab^	7.27 ^Aa^	0.18	<0.01	<0.01	<0.01
	V-YX	2.56 ^Bb^	3.04 ^Cab^	3.15 ^Dab^	3.69 ^BCa^				
	V-493	3.05 ^Bb^	3.49 ^Cb^	4.01 ^Cab^	4.28 ^Ba^				
	NV-CK	4.17 ^Ac^	4.68 ^Bbc^	5.33 ^Bb^	6.82 ^Aa^				
	NV-YX	2.74 ^B^	3.05 ^C^	3.15 ^D^	3.36 ^C^				
	NV-493	2.60 ^Bc^	3.37 ^Cb^	4.01 ^Cab^	4.42 ^Ba^				
PA (g/kg DM)	V-CK	ND	ND	ND	13.11	−	−	–	–
	V-YX	ND	ND	ND	ND				
	V-493	ND	ND	ND	ND				
	NV-CK	ND	ND	ND	12.44				
	NV-YX	ND	ND	ND	ND				
	NV-493	ND	ND	ND	ND				
BA (g/kg DM)	V-CK	ND	ND	ND	11.53	–	–	–	−
	V-YX	ND	ND	ND	ND				
	V-493	ND	ND	ND	ND				
	NV-CK	ND	ND	ND	12.17				
	NV-YX	ND	ND	ND	ND				
	NV-493	ND	ND	ND	ND				

Dry matter, DM; lactic acid, LA; acetic acid, AA; propionic acid, PA; butyric acid, BA; ammonia nitrogen, NH_3_-N; control, CK; vacuum treatment, V; inoculated with YX, YX; inoculated with *Lactobacillus plantarum* ZZUA493, 493; nonvacuum treatment, NV; treatments T; ensiling days, D; the interaction between T and D, T × D; standard error of mean, SEM; not detected, ND. Means with different letters in the same row (a–c) or column (A–D) differ (*p* < 0.05).

**Table 3:** Alpha diversity of bacterial diversity at 90 days of ensiling.

Samples	Observed	Chao1	ACE	Shannon	Simpson	Coverage
V-CK	182.00	265.92	307.92	1.73 ^a^	0.26 ^d^	0.9994
V-YX	151.33	335.73	324.88	0.36 ^d^	0.86 ^a^	0.9993
V-493	204.67	371.37	373.85	0.85 ^c^	0.58 ^b^	0.9992
NV-CK	180.50	254.22	268.60	1.45 ^b^	0.36 ^c^	0.9995
NV-YX	151.67	257.28	286.22	0.43 ^d^	0.84 ^a^	0.9994
NV-493	208.00	366.84	369.41	1.29 ^b^	0.42 ^a^	0.9992

Control, CK; vacuum treatment, V; inoculated with YX, YX; inoculated with *Lactobacillus plantarum* ZZUA493, 493; nonvacuum treatment, NV. Different lowercase letters indicate a significant difference between samples (*p* < 0.05).

**Table 4:** Effects of vacuum treatment, inoculants and their interactions on the relative abundance of genera at 90 days.

Relative Abundance (%)	Vacuum				Nonvacuum		SEM	*p*-Value		
	V-CK	V-YX	V-493	NV-CK	NV-YX	NV-493		V	T	V × T
*Pediococcus*	30.06 ^b^	92.39 ^a^	21.85 ^b^	10.67 ^c^	91.39 ^a^	27.67 ^b^	0.03	NS	<0.01	NS
*Lactobacillus*	3.67 ^cd^	6.04 ^c^	73.90 ^a^	1.31 ^d^	5.25 ^cd^	60.06 ^b^	0.12	<0.01	<0.01	<0.01
*Garciella*	26.43 ^b^	<0.001 ^c^	< 0.001 ^c^	51.73 ^a^	<0.001 ^c^	0.07 ^c^	0.23	<0.01	<0.01	<0.01
*Enterococcus*	29.43 ^a^	0.33 ^b^	0.84 ^b^	28.55 ^a^	0.52 ^b^	2.95 ^b^	0.12	NS	<0.01	NS
*Enterobacter*	0.32 ^c^	0.51 ^c^	1.77 ^b^	1.41 ^b^	1.73 ^b^	6.13 ^a^	0.06	<0.01	<0.01	<0.05
*Staphylococcus*	4.69 ^a^	0.04 ^b^	0.04 ^b^	3.43 ^a^	0.10 ^b^	0.18 ^b^	0.09	NS	<0.01	NS
*Corynebacterium*	1.74	<0.001	ND	0.08	ND	<0.001	0.07	NS	NS	NS

Control, CK; vacuum treatment, V; inoculated with YX, YX; inoculated with *Lactobacillus plantarum* ZZUA493, 493; nonvacuum treatment, NV; treatments, T; interaction between V and T, V × T; standard error of mean, SEM; not significant, NS; not detected, ND. Different lowercase letters indicate significant differences in relative abundance of the same genus among different treatments (*p* < 0.05).

**Table 5:** Mantel test and Spearman’s correlation analysis for fermentation characteristics of alfalfa silage and relative abundance of bacteria at the genus level.

	pH		LA		AA		LA/AA		WSC		NH_3_-N		BA	
	*r*	*p*	*r*	*p*	*r*	*p*	*r*	*p*	*r*	*p*	*r*	*p*	*r*	*p*
**Mantel test**														
*Pediococcus*	−0.500	0.043	0.137	0.599	0.123	0.639	0.199	0.443	0.168	0.518	−0.752	0.001	−0.323	0.206
*Lactobacillus*	−0.539	0.028	0.392	0.120	0.630	0.008	0.012	0.966	0.741	0.001	−0.238	0.357	−0.553	0.021
*Garciella*	0.783	0.000	−0.420	0.093	−0.424	0.090	−0.117	0.655	−0.610	0.009	0.629	0.007	0.624	0.007
*Enterococcus*	0.914	0.000	−0.201	0.438	−0.392	0.120	−0.025	0.928	−0.282	0.274	0.863	0.000	0.724	0.001
*Enterobacter*	0.056	0.831	−0.436	0.082	0.216	0.404	−0.510	0.039	0.278	0.280	−0.027	0.921	−0.491	0.046
*Staphylococcus*	0.824	0.000	−0.350	0.168	−0.429	0.087	−0.056	0.831	−0.483	0.049	0.725	0.001	0.629	0.007
*Corynebacterium*	0.620	0.008	−0.072	0.783	−0.234	0.366	0.041	0.875	−0.496	0.043	0.746	0.001	0.661	0.004
**Spearman’s correlation analysis**														
Chao1	−0.328	0.198	0.549	0.024	0.184	0.479	0.353	0.165	−0.574	0.016	−0.002	0.996	−0.296	0.248
Shannon	0.816	0.000	−0.037	0.891	−0.279	0.276	0.103	0.694	−0.269	0.296	0.941	0.000	0.718	0.001

LA, lactic acid; AA, acetic acid; LA/AA, lactic to acetic ratio; WSC, water-soluble carbohydrate; NH_3_-N, ammonia nitrogen; BA, butyric acid.

## Data Availability

Data are contained within the article.
